# Maternal docosahexaenoic acid supplementation shapes offspring gut microbiota to modulate the gut-brain axis in a sow-piglet model

**DOI:** 10.3389/fnut.2026.1776896

**Published:** 2026-04-13

**Authors:** Soon Lee, Stephanie Dubrof, Ishfaque Ahmed, Qun Zhao, Todd R Callaway, Jeferson Lourenco, Hea Jin Park

**Affiliations:** 1 Department of Nutritional Sciences, College of Family and Consumer Sciences, University of Georgia, Athens, GA, United States; 2 Department of Physics and Astronomy, Franklin College of Arts and Sciences, University of Georgia, Athens, GA, United States; 3 Department of Animal and Dairy Science, College of Agricultural and Environmental Sciences, University of Georgia, Athens, GA, United States

**Keywords:** docosahexaenoic acid, gut microbiome, gut-brain axis, infant brain development, maternal nutrition

## Abstract

**Introduction:**

Modification of maternal nutrition during the perinatal period represents an important window that may influence offspring neurodevelopment. Docosahexaenoic acid (DHA), a key omega-3 polyunsaturated fatty acid found in the brain, is reported to have beneficial effects on cognitive outcomes of infants. However, its specific effects on the shaping of gut microbiota to influence the piglet gut-brain axis remain to be elucidated.

**Methods:**

Using a sow-piglet model, this study aimed to investigate changes in offspring gut microbiota, intestinal barrier integrity, and their correlations with brain resting-state functional connectivity following maternal supplementation of DHA.

**Results:**

Piglets born to DHA-supplemented sows showed significant differences in microbial alpha- and beta-diversity compared to control piglets. Jejunal claudin-1 expression was upregulated in DHA piglets, and tight junction protein levels were positively correlated with specific microbial taxa. Furthermore, gut microbial diversity and specific taxa were significantly associated with functional brain networks.

**Discussion:**

Our findings demonstrate the role of maternal DHA supplementation in shaping offspring gut microbiome and gut integrity, potentially altering offspring brain function networks. Furthermore, these results underscore the importance of gut microbiota shaping through perinatal nutritional interventions as a means of programming the gut-brain axis in the early stages of life.

## 1 Introduction

The nutritional status of infants during development relies heavily on maternal diet during the perinatal stage, which lays the foundation of their health in the long-term. The major developmental phases of the brain begins *in utero* and continues up to around 3 years after birth (1), making this a critical period particularly susceptible to inappropriate nutritional status (2, 3). Docosahexaenoic acid (DHA) is an omega-3 polyunsaturated fatty acid (PUFA) predominantly found in the brain, often taken as a supplement by pregnant and lactating mothers. Existing human studies indicate a positive correlation between maternal supplementation or status of DHA and their offspring’s cognitive development, including memory (4), attention (5) and problem-solving abilities (6). We have previously reported that perinatal supplementation of DHA led to changes in brain resting-state functional connectivity of offspring at weaning (7, 8), potentially via a novel class of PUFA metabolites called specialized pro-resolving mediators (9), supporting the idea that DHA status during early life is associated with cognitive development.

The gut-brain axis has emerged as a bidirectional communication network bridging the central and the enteric nervous system, through changes in the gut microbiota and its metabolites (10–16). Microbial derived short-chain fatty acids, including acetate, propionate and butyrate, modulate blood–brain barrier integrity, inflammation, proliferation of neural progenitor cells, and microglial maturation (10–13). They also interact with neurotransmitters such as serotonin, dopamine, norepinephrine, and gamma-aminobutyric acid (GABA), tryptophan metabolites, and bile acids to regulate the gut and brain crosstalk (13–15). Importantly, the critical window of neurodevelopment coincides with the growing diversity and richness of the gut microbiota (17–19), and studies suggest that this colonization of microbiota may act as an essential signal for the gut-brain axis (18, 20). Animal studies have shown that changes in the microbial population of offspring are closely associated to their development in neuroplasticity (21) and neurotransmission (22), as well as behavioral outcomes, such as social deficits (21), learning, and memory (23, 24). The shaping of the gut microbiota is influenced by various dietary (25), environmental (25–27), and maternal sources (7, 27, 28), which may initiate the microbial colonization in infants from as early as gestation (29, 30). Alterations in infant gut microbiota following fish oil or DHA intake in mothers have been observed, urging the need for further investigation into how DHA supplementation may regulate the gut-brain axis to benefit offspring development. Human trials have shown that maternal DHA or fish oil intake during pregnancy or lactation may influence the diversity (31) and abundance of certain taxa such as *Lactobacillus* and *Bifidobacterium* in infants (31, 32). Maternal DHA or n-3 PUFA supplement in mice led to shifts in microbial richness (33) and changes in *Ruminococcus*, *Lactobacillus*, *Barnesiella* (33), *Bacteroides*, *Akkermansia* and *Epsilonproteobacteria* abundances in offspring (34). Thus, while results across human and animal studies report that maternal DHA intake may shape offspring gut microbiome, further studies are required to explore the DHA-induced changes in the gut microbiota and gut-brain axis in offspring following maternal supplementation.

Building on our previous findings on the beneficial effect of perinatal DHA supplementation on offspring (7), we aimed to explore the effect of maternal DHA intake on fecal microbiota and its association with functional connectivity in the brain of offspring. A sow-piglet model was utilized in this study due to their brain anatomy (35, 36) and the pattern of growth spurt that resembles that of human brains (37, 38). Further, pigs share a similar intestinal anatomy and physiology (39), and gut microbiota geography with humans (40, 41), making them highly translatable models in nutritional research. Despite evidence that maternal DHA supplementation may modulate offspring brain development and the gut environment, it remains unclear how DHA-induced modulations are linked to the activation of functional brain networks and changes in intestinal integrity, particularly in a large animal model. Therefore, this study aimed to explore the effects of maternal DHA supplementation in shaping the gut microbiota of offspring, intestinal tight junction protein levels and brain resting-state functional connectivity, to better understand its role in programming the early life gut-brain axis.

## 2 Materials and methods

### 2.1 Animal handling and study design

All animal handling and sampling procedures were approved by the University of Georgia Animal Care and Use Committee. The NIH’s guide for the Use and Care of Laboratory Animals (AUP number: A2021 01–026) was followed. Crossbreed commercial Landrace sows (*n* = 12) were artificially inseminated at the University of Georgia Swine Unit and transferred to the Large Animal Research Unit animal facility at 60 to 65 days of gestation. Sows were allocated to the control group (*n* = 6) or DHA group (*n* = 6) according to body weight (239.3 ± 7.6 kg; mean ± SEM) and parity (4.0 ± 0.2; mean ± SEM). Sows in the DHA group were supplemented with 75 mg/kg of bodyweight/day of DHA derived from algae (contains 44.6% DHA, life’s DHA S40, DSM Nutritional Products, Inc., Kingstree, SC, USA). The dosage of DHA treatment was calculated based on existing research showing beneficial effects of DHA on pregnant sows and their offspring (7, 42). Both groups were fed 2 kg of gestation diet per sow per day, followed by *ad libitum* access to a lactation diet post-farrowing. At farrowing, 2 piglets from each litter (1 male and 1 female) with body weights closest to the average of their respective litter were selected. After fecal sample collection and functional magnetic resonance imaging (fMRI) at weaning (postnatal day 19–21), the piglets were sacrificed, and their jejunum mucosal content was scraped using sterile materials. Fecal samples were collected from the piglets at weaning (PND18) by stimulation using sterilized cotton swabs. Instantly upon defecation, the stool was collected directly into a sterile tube, ensuring no contact with the piglet’s body or surrounding surfaces. The collected samples were immediately frozen and stored at −80 °C until further analysis.

### 2.2 Microbial DNA extraction and 16S rRNA gene sequencing

Microbial DNA was extracted from the fecal samples following a methodology previously described, which outlines an efficacious approach by combining mechanical and enzymatic techniques (43). In short, a modified version of the QIAamp Fast DNA Stool Mini Kit (QIAGEN Sciences, Germantown, MD, USA) utilizing Lysing Matrix E tubes (MP Biomedicals, Irvine, CA, USA) were used to process 330 mg of fecal material. At the end of the process, purified DNA was eluted in 100 μL of buffer and quantified using the Synergy H4 Hybrid Multi-Mode Microplate Reader (Agilent Technologies, Santa Clara, CA, USA). The 16S ribosomal RNA (rRNA) gene was sequenced at a commercial lab (LC Sciences, LLC, Houston, TX, USA) on an Illumina NovaSeq 6000 platform (Illumina, San Diego, CA, USA). The forward primer S-D-Bact-0341-b-S-17 (5′-CCTACGGGNGGCWGCAG-3′) and reverse primers S-D-Bact-0785-a-A-21 (5′-GACTACHVGGGTATCTAATCC-3′) were used to amplify the V3-V4 region of the 16S rRNA gene (44). Paired-end FASTQ files were demultiplexed and imported into QIIME 2 v.2023.2 (45), and the DADA2 plugin (46) was used to control sequence quality, merge reads, and remove chimeras. The generated amplicon sequence variants (ASV) were filtered, so only ASVs with frequency equal or greater than two were retained. Those ASVs were then classified using a Naïve Bayes classifier (47) trained on the Greengenes2 reference database (48). Taxonomic data were cleaned and summarized using the mbX package in R (49). Microbial taxa were summarized as relative abundance at different taxonomic levels, ranging from phyla to species. Diversity analysis was performed at the ASV level, with samples being rarefied to a common sequencing depth of 33,481 sequences/sample for calculation of alpha diversity (microbial richness, diversity, and evenness) and beta diversity (Unweighted and Weighted UniFrac distances) (50).

### 2.3 Immunoblotting

For western blot analysis, the jejunum tissues were mixed with RIPA Lysis Buffer System (sc-24948A, Santa Cruz Biotechnology, USA) and homogenized at 15,000 RPM for 25 s. The protein concentration was determined using the BCA Protein Assay Kit (#23225, Pierce, USA) and proteins were denatured at 95 °C for 5 min. Electrophoresis was run on tris-glycine gels (XP10200BOX, ThermoFisher Scientific, USA) at 60 V for 30 min and 100 V for 2 h and the blot was transferred to nitrocellulose membranes (IB23001, ThermoFisher Scientific, USA) using iBlot™2 Dry Blotting System (IB21001, Invitrogen, USA). The membranes were blocked at room temperature for 1 h and incubated overnight at 4 °C with 1:500 diluted primary antibodies against claudin (EPRR1887, Abcam, USA), occludin (AB31721, Abcam, USA), ZO-1 (AB27613, Abcam, USA), and 1:2000 dilution of *β*-actin (A5441, Sigma-Aldrich, USA). HRP-conjugated secondary antibodies, anti-mouse (926-80010, LICORbio, USA) or anti-rabbit (926-80011, LICORbio, USA) diluted to 1:2000, were attached before visualization by Chemidoc MP system (Bio-Rad, USA) with Immobilon Classico Western HRP Substrate (WBLUC0100, Sigma-Aldrich, USA). The intensities of the bands were quantified using ImageJ (NIH, USA).

### 2.4 Statistical analysis

A linear mixed-effect model was employed to assess treatment effects after controlling for sex as a fixed effect and for maternal as a random effect. RStudio (Version 2022.07.2 + 576, R Foundation for Statistical Computing, Vienna, Austria) and GraphPad Prism 10.5 (GraphPad Software, USA) was used for statistical analysis and visualization of the data. Relative abundance of individual taxa was analyzed using ANCOM-BC2 (51) with sow as a random factor in the models. Beta-diversity was analyzed using the ADONIS permutation-based statistical test in vegan-R, using sow as a block. Unpaired *t*-tests were employed to compare the alpha diversity indices and the protein expression levels between the control and DHA groups. Correlation analysis was performed to assess the relationship between gut microbiota and different outcome measures, including protein expression levels and resting-state networks. For correlation analysis including microbiome data, individual taxa with a mean relative abundance of ≥0.01% at the phylum level and ≥0.1% at the family and genus levels were included. Outliers falling outside mean ± 2 standard deviations were excluded. *p* < 0.05 was considered statistically significant. The data are represented as means ± the standard error of the mean (SEM).

## 3 Results

### 3.1 Maternal supplementation of DHA increased the fecal microbiota diversity of offspring at weaning

**Figure 1:**
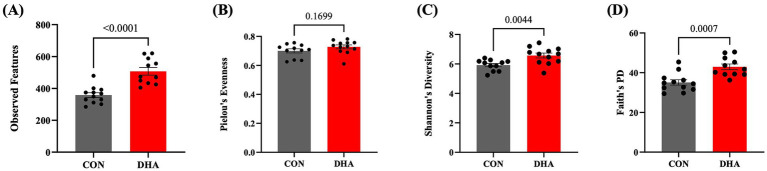
Effect of maternal DHA supplementation on alpha diversity of piglet gut microbiota at weaning. Alpha diversity analysis of piglet gut microbiota at weaning following maternal DHA supplementation. Indices **(A)** observed features, **(B)** Pielou’s evenness, **(C)** Shannon’s diversity, and **(D)** Faith’s PD were calculated. The data are shown as means ± SEM. CON: control; DHA: docosahexaenoic acid; PD: phylogenetic diversity.

**Figure 2:**
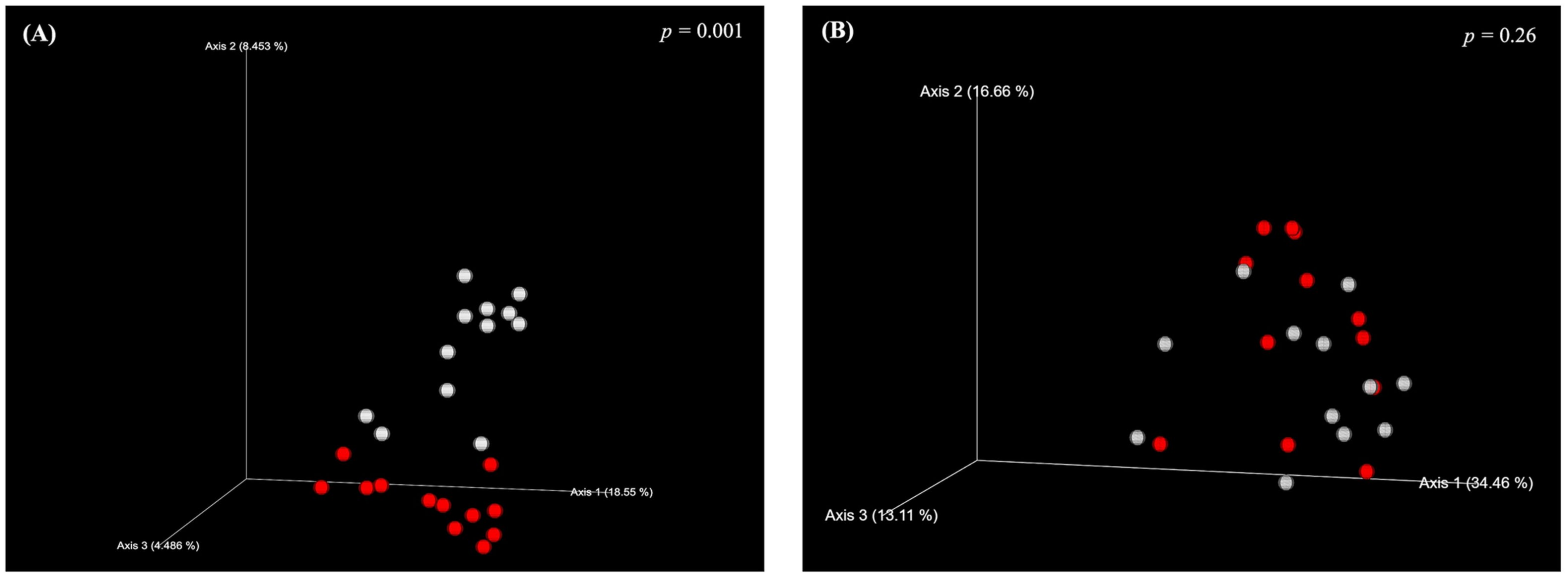
Effect of maternal DHA supplementation on beta diversity of piglet gut microbiota at weaning. Beta diversity analysis of piglet gut microbiota at weaning following maternal DHA supplementation: **(A)** Unweighted and **(B)** weighted UniFrac was used for calculation. White dots represent control piglets, and red plots represent DHA piglets.

Various measures of alpha diversity in piglets at weaning were calculated to assess the changes in within sample diversity of piglet microbiome in response to perinatal DHA supplementation (Figure 1). Compared to control piglets, the piglets of DHA-supplemented sows showed higher alpha diversity measures at weaning. A greater species richness was found in DHA piglets, as evidenced by their higher number of Observed Features by 34.58% (*p* < 0.0001) compared to control (Figure 1A). DHA pigs also presented higher Shannon’s Diversity by 10.39% (*p* = 0.0044) (Figure 1C) and Faith’s phylogenetic diversity index by 20.14% (*p* = 0.0007) (Figure 1D). The two groups did not display any differences in Pielou’s evenness index, which simply indicates the evenness of distribution in a microbial community (Figure 1B). The impact of DHA supplementation on beta-diversity was measured using both the Unweighted and Weighted UniFrac methods to assess the similarity of microbial patterns among groups. A significant difference between control piglets and DHA piglets was observed in Unweighted (*p* = 0.001), but not for Weighted (*p* = 0.26) UniFrac at weaning (Figures 2A,B).

### 3.2 Maternal supplementation of DHA altered the gut microbiota population of piglets at weaning

**Figure 3:**
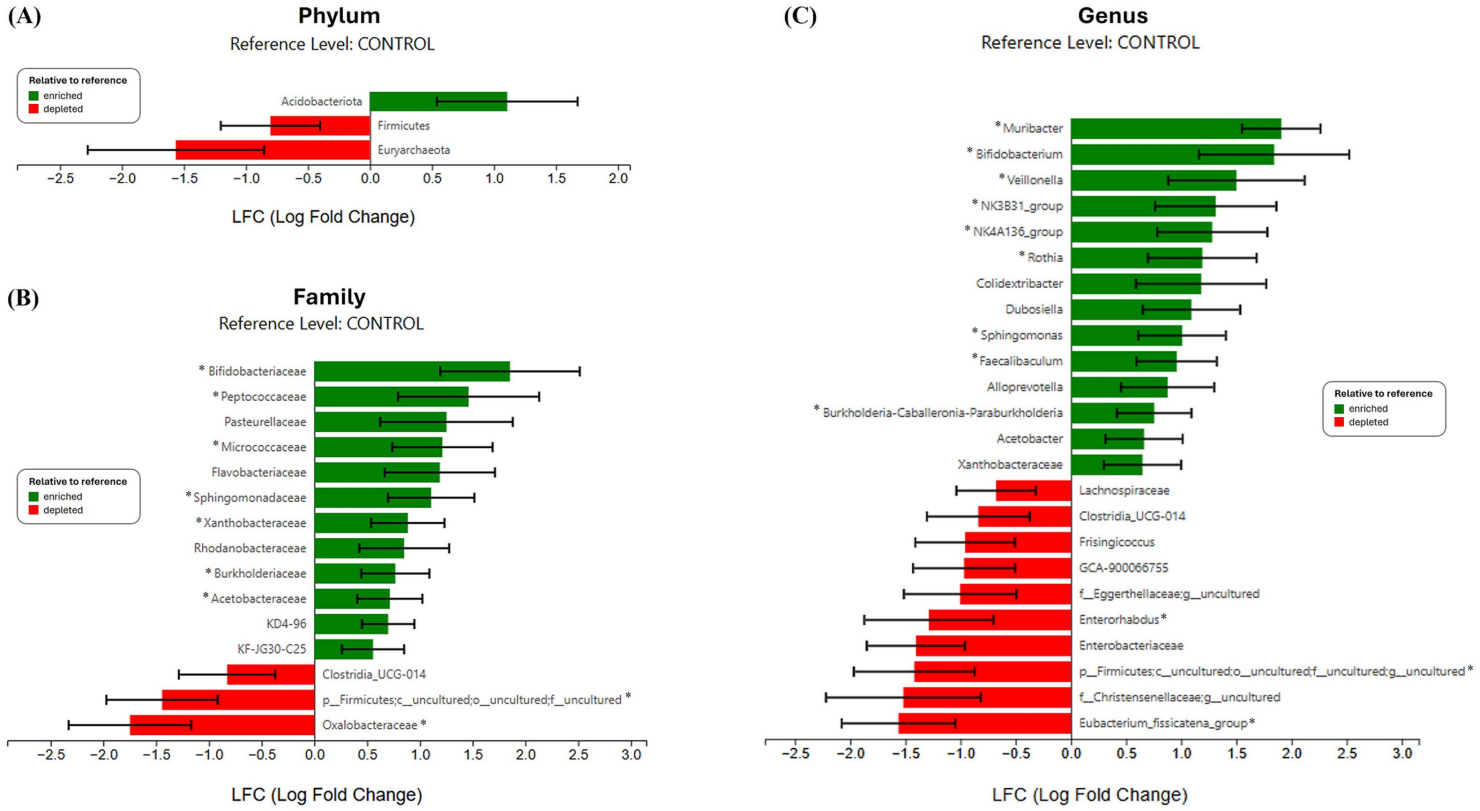
ANCOM-BC2 analysis of DHA maternal supplementation on offspring gut microbiome. Differentially abundant microbial taxa at the **(A)** phylum, **(B)** family, and **(C)** genus levels associated with DHA maternal supplementation based on ANCOM-BC2 analysis. Green bars indicate taxa enriched in piglets in DHA piglets, while red bars indicate depleted taxa depleted in DHA piglets. Only taxa with significant (*p < 0.05) or trend of differential abundance (*p* < 0.1) are shown.

The differential abundance of piglet gut microbiota at weaning were analyzed by ANCOM-BC2 (Figure 3). At the phylum level, three taxa were identified to have a trend for differential abundance between piglets born to control or DHA supplemented sows. Specifically, *Acidobacteriota* was enriched (*p* = 0.0826), and *Firmicutes* (*p* = 0.0729) and *Euryarchaeota* (*p* = 0.0536) were depleted in DHA piglets compared to control piglets (Figure 3A). At the family level, 15 different taxa were identified to have a significant or a trend of difference between control and DHA piglets, with 12 families enriched in DHA piglets compared to control piglets (Figure 3B). Families that were significantly enriched in DHA gut microbiome were *Bifidobacteriaceae* (*p* = 0.0187), *Peptococcaceae* (*p* = 0.046), *Micrococcaceae* (*p* = 0.03), *Sphingomonadaceae* (*p* = 0.0277), *Xanthobacteraceae* (*p* = 0.0295), *Burkholderiaceae* (*p* = 0.0375), and *Acetobacteraceae* (*p* = 0.0301). *Oxalobacteraceae* (*p* = 0.0298) was identified to be depleted in DHA piglets compared to control piglets. Finally at the genus level, 24 different taxa were identified to have a significant or a trend of difference between control and DHA piglets, with 14 genera enriched in DHA piglets compared to control piglets (Figure 3C). Genera that were significantly enriched in DHA piglets compared to control piglets included *Muribacter* (*p* = 0.0059), *Bifidobacterium* (*p* = 0.022), *Veillonella* (*p* = 0.0379), NK3B31 (*p* = 0.039), NK4A136 (*p* = 0.029), *Rothia* (*p* = 0.0377), *Sphingomonas* (*p* = 0.0393), and *Faecalibaculum* (*p* = 0.0249). On the other hand, *Enterorhabdus* (*p* = 0.0277) and *Eubacterium fissicatena* (*p* = 0.0277) were significantly depleted in DHA piglets compared to CON piglets.

### 3.3 Maternal supplementation of DHA increased tight junction protein levels in piglet jejunum

**Figure 4:**
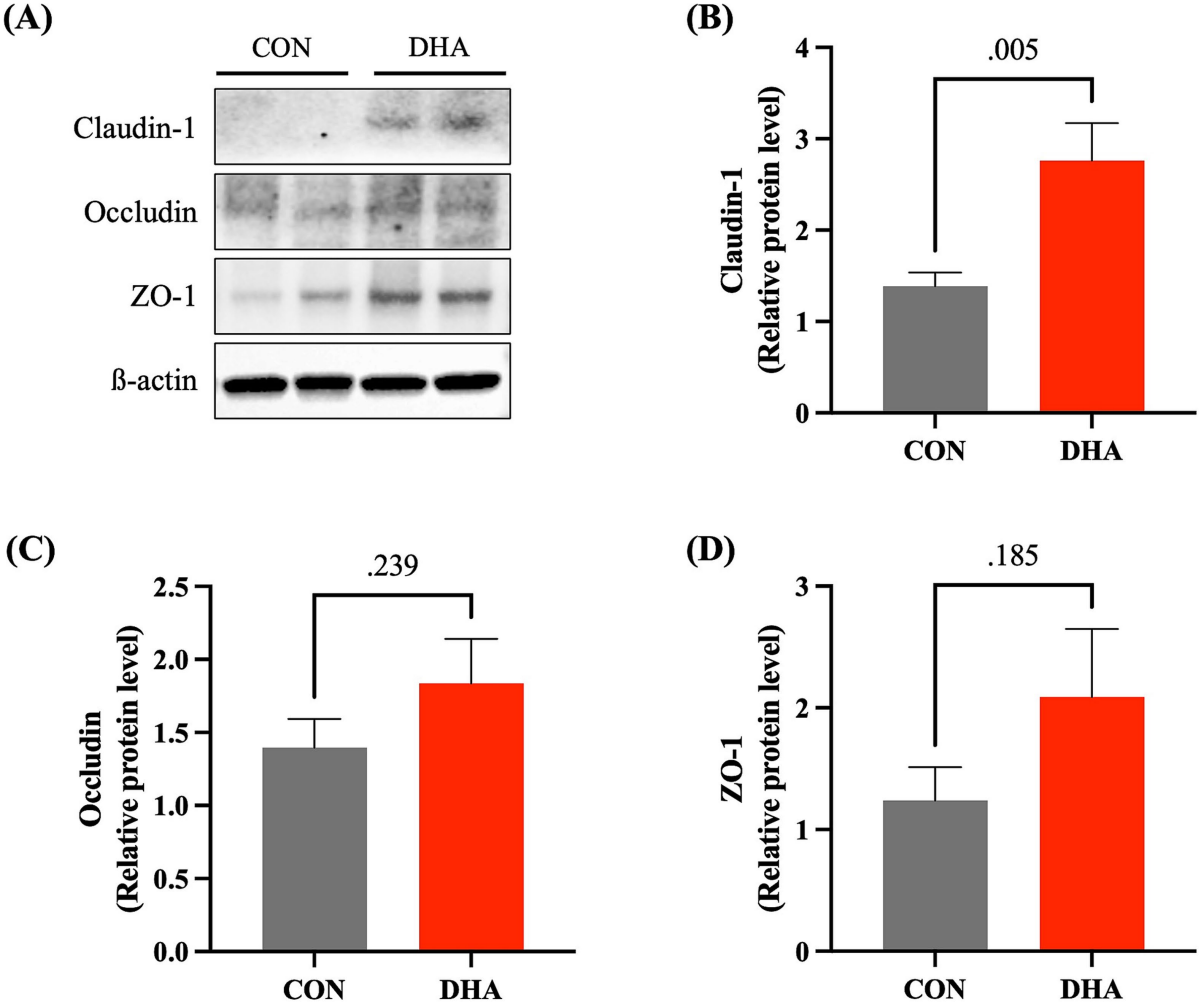
Expression levels of tight junction proteins in piglet jejunum at weaning. **(A)** Representative images of western blots of tight junction proteins in piglet jejunum. Band density of **(B)** Claudin-1, **(C)** Occludin, and **(D)** ZO-1 was quantified using Image J and normalized against ß-actin. The data are shown as means ± SEM. CON: control; DHA: docosahexaenoic acid.

Tight junction proteins in the intestinal epithelial cells are essential for maintaining physical barrier function, and downregulation of their expression levels indicate elevated gut permeability (52). The pig jejunum takes up approximately 80% of their small intestines (53), making it one of the major areas that may experience inflammation and oxidative stress potentially leading to disruptions in gut barrier integrity (54). Thus, we measured the changes in the expressional levels of tight junction proteins in the jejunum of piglets following maternal supplementation of DHA (Figure 4). DHA piglets showed a significant increase (*p* = 0.005) of over 2-folds in claudin-1 protein levels compared to control (Figure 4A). The increases in the levels of occludin and ZO-1 following maternal supplementation did not appear to be statistically significant (Figures 4B,C).

### 3.4 Tight junction protein levels in piglet jejunum were associated with specific taxa of gut microbiota

**Figure 5:**
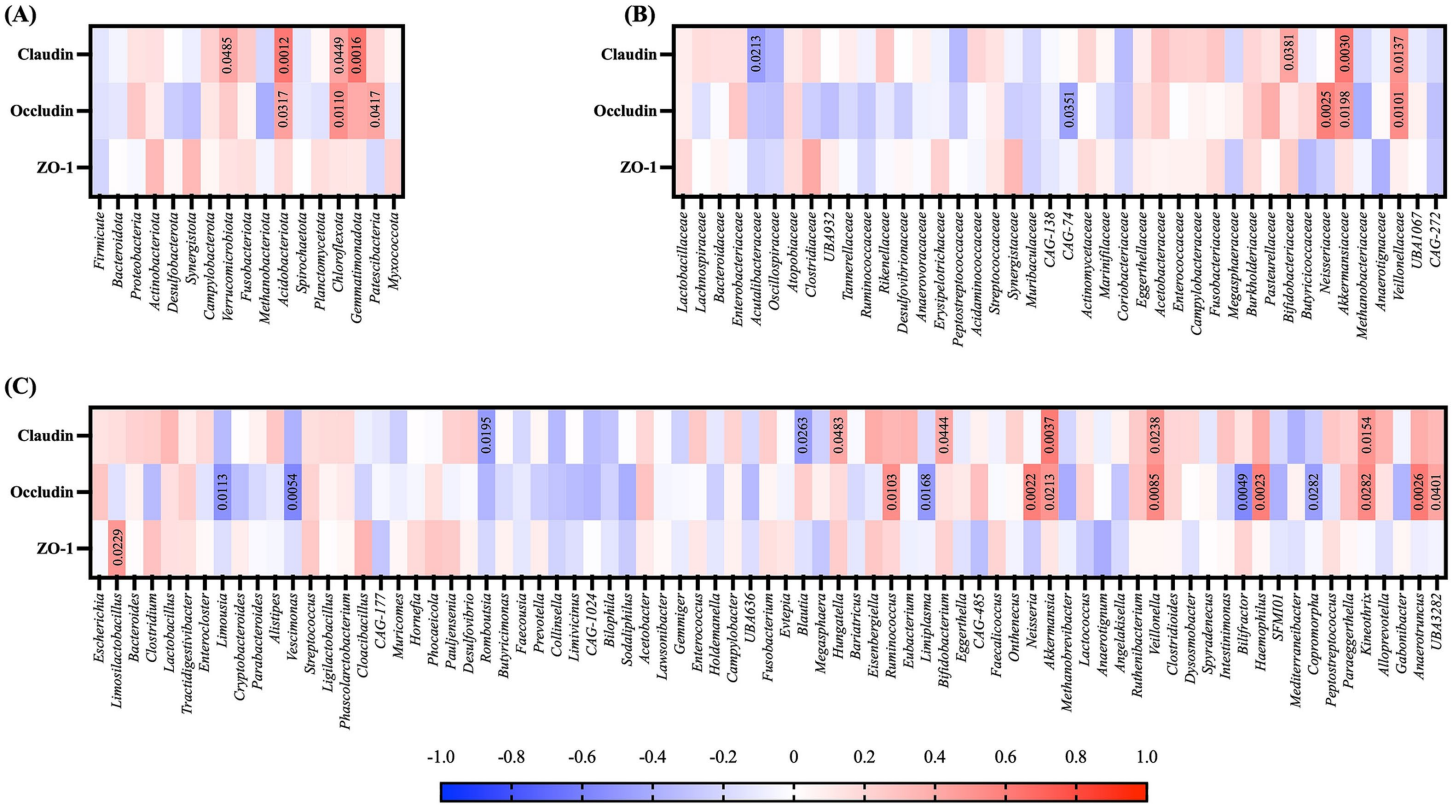
Correlation heatmap of tight junction proteins with gut microbiota of piglets at weaning. Correlation analysis of tight junction protein levels with the relative abundance of gut microbiota at the **(A)** phylum, **(B)** family, and **(C)** genus level. Spearman’s rank correlation analysis was performed. *p* < 0.05 are shown in the corresponding boxes.

The correlation of the levels of tight junction proteins in piglet jejunum with gut microbiota were analyzed (Figure 5). At the phylum level, expression of claudin-1 was positively correlated with the abundance of *Verrucomicrobiota* (*p* = 0.0485), *Acidobacteriota* (*p* = 0.0012), *Chloroflexota* (*p* = 0.0449), and *Gemmatimonadota* (*p* = 0.0016). Occludin also showed a significant positive correlation with *Acidobacteriota* (*p* = 0.0317), *Chloroflexota* (*p* = 0.011), and *Patescibacteria* (*p* = 0.0417) (Figure 5A). At the family level, claudin displayed a positive correlation with *Bifidobacteriaceae* (*p* = 0.0381), *Akkermansiaceae* (*p* = 0.003), and *Veillonellaceae* (*p* = 0.0137) and a negative correlation with *Acutalibacteraceae* (*p* = 0.0213) Occludin was positively correlated with *Neisseriaceae* (*p* = 0.0025), *Akkermansiaceae* (*p* = 0.0198), and *Veillonellaceae* (*p* = 0.0101) and negatively correlated with CAG-74 (*p* = 0.0351). Finally, at the genus level, all three proteins measured showed a significant correlation with the abundance of specific microbiota taxa. The level of claudin was positively correlated with *Hungatella* (*p* = 0.0483), *Bifidobacterium* (*p* = 0.0444), *Akkermansia* (*p* = 0.0037), *Veillonella* (*p* = 0.0238), and *Kineothrix* (*p* = 0.0154) and negatively correlated with *Romboutsia* (*p* = 0.0195) and *Blautia* (*p* = 0.0263). Occludin level was positively correlated with *Ruminococcus* (*p* = 0.0103), *Neisseria* (*p* = 0.0022), *Akkermansia* (*p* = 0.0213), *Veillonella* (*p* = 0.0085), *Haemophilus* (*p* = 0.0023), *Kineothrix* (*p* = 0.0282) *Anaerotruncus* (*p* = 0.0026) and UBA3282 (*p* = 0.0401), and negatively correlated with *Limousia* (*p* = 0.0113), *Vescimonas* (*p* = 0.0054), *Limiplasma* (*p* = 0.0168), *Bilifractor* (*p* = 0.0049) and *Copromorpha* (*p* = 0.0282). Finally, the level of ZO-1 was correlated with *Limosilactobacillus* (*p* = 0.0229) (Figure 5C).

### 3.5 Piglet gut microbiota was associated with the activity of cerebellum, visual and salience network of brain in offspring at weaning

**Figure 6:**
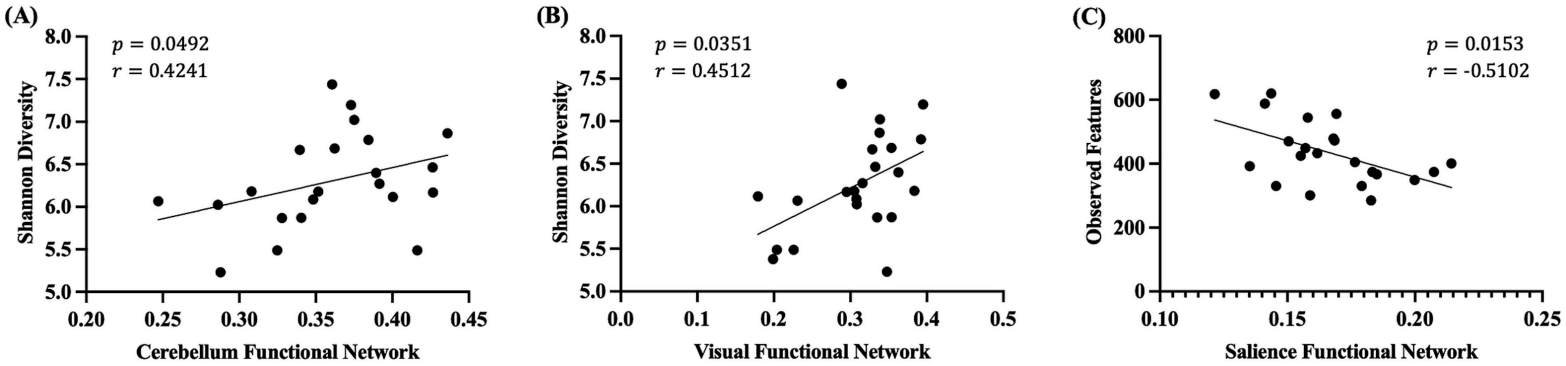
Correlation analysis between alpha diversity of gut microbiota and resting state networks of piglets at weaning. Correlation analysis between four alpha diversity indices and 7 different resting-state functional networks were performed. Only those with a statistically significant difference (*p* < 0.05) are shown. The scatter plots display the correlations for **(A)** Pielou’s Evenness and **(B)** Shannon’s Diversity with the visual functional network, and **(C)** Faith’s PD and **(D)** Observed Features with the salience functional network. Spearman’s rank correlation analysis was performed. The correlation coefficient *r* and corresponding *p* values are noted within each graph. PD: Phylogenetic diversity.

**Figure 7:**
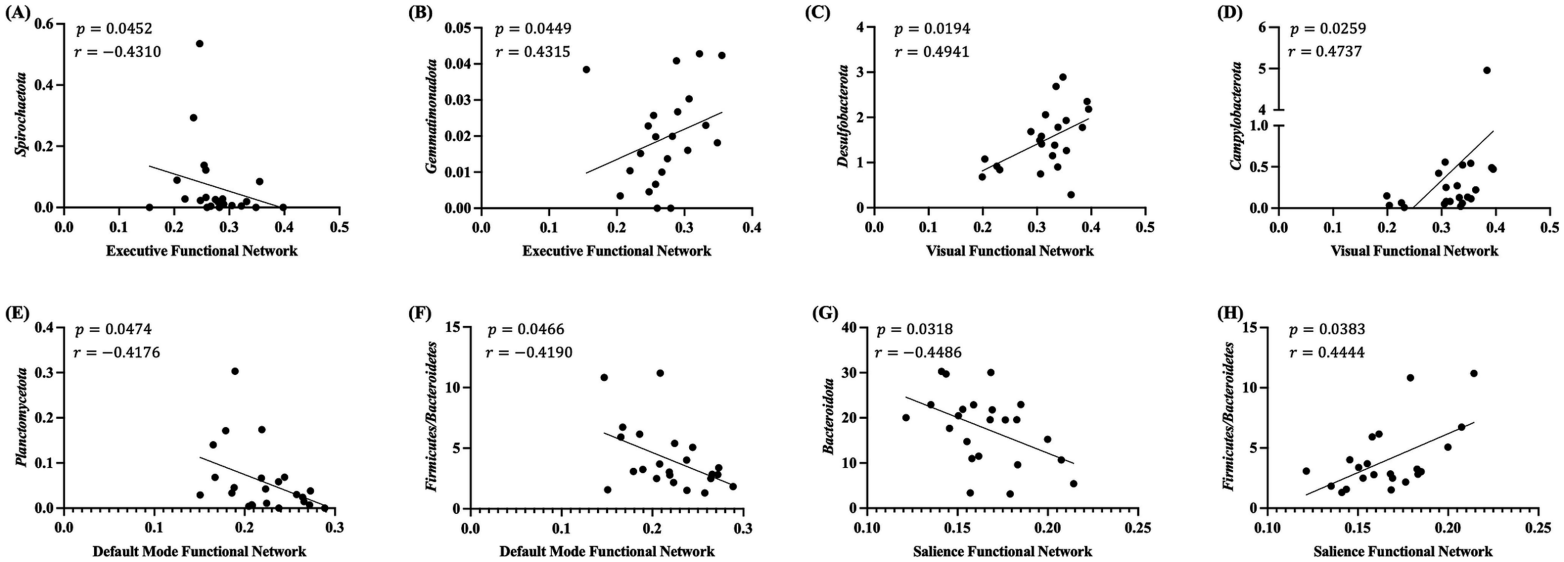
Correlation analysis between gut microbiota and resting state networks of piglets at weaning. Correlation analysis between gut microbiota taxa at the phylum level and different functional networks were performed. Only those with a statistically significant difference (*p* < 0.05) are shown. The scatter plots display correlations for **(A)** *Campylobacterota* with the executive network, **(B)** *Desulfobacterota* with the visual network, **(C)** *Bacteroidota* with the salience network, and the F/B ratio with the **(D)** default mode and **(E)** salience networks. Spearman’s rank correlation analysis was performed. The correlation coefficient *r* and corresponding *p* values are noted within each graph. *F/B: Firmicutes/Bacteroidetes*.

Previously, we identified that piglets born from DHA-supplemented sows had altered connectivity in different resting-state functional networks, including the cerebellar, visual, default mode, executive control and sensorimotor network (7). Thus, a correlation analysis of piglet brain resting-state functional connectivity with their gut microbiota at weaning was performed. The activation of several resting-state functional networks was significantly correlated with measures of alpha diversity (Figure 6). Shannon’s diversity was positively correlated with cerebellum network (*p* = 0.0492, *r* = 0.4241) (Figure 6A) and visual network (*p* = 0.0351, *r* = 0.4512) (Figure 6B) activation. On the other hand, salience network was negatively correlated with the number of observed features (*p* = 0.0153, *r* = −0.5102) (Figure 6C). Four different resting-state functional networks were significantly correlated with specific gut microbiota taxa at the phylum level (Figure 7). Executive network was negatively correlated with *Spirochaetota* (*p* = 0.0452, *r* = −0.4310) and positively correlated with *Gemmatimonadota* (*p* = 0.0449, *r* = 0.4315) (Figures 7A,B). Visual network had a positive correlation with *Desulfobacterota* (𝑝 = 0.0194, *r* = 0.4941) and *Campylobacterota* (𝑝 = 0.0259, *r* = 0.4737) (Figures 7C,D). Default mode network was negatively correlated to *Planctomycetota* (𝑝 = 0.0474, *r* = −0.4176) and the *Firmicutes* to *Bacteroidetes* ratio (𝑝 = 0.0466, *r* = −0.4190) (Figures 7E,F). Lastly, salience network was negatively correlated to *Bacteroidota* (𝑝 = 0.0318, *r* = −0.4486) and positively correlated to *Firmicutes* to *Bacteroidetes* (𝑝 = 0.0383, *r* = 0.4444) ratio (Figures 7G,H).

Table 1 shows the different resting state networks and the individual taxa of microbiota that had a significant correlation to each of these networks, at the family and genus level. At the family level, the visual functional network had the highest number of individual taxa that were significantly correlated (5 positive). Significant correlations were also shown in the executive, cerebellum, sensorimotor, default mode and salience network. At the genus level, the sensorimotor network had the highest number of correlations (1 positive and 10 negative). The executive, default mode, and basal ganglia network only showed negative correlations with the genus level taxa. Interestingly, the default mode network did not display any significant correlation with the individual taxonomic groups of gut microbiomes analyzed at the family and genus level. Contrary to the family level, correlations were also found in the auditory and basal ganglia networks at the genus level.

**Table 1:** Summary of significant correlations between gut microbiota taxa and resting-state functional networks of piglets at weaning.

Network	Family	Genus
Executive functional network	*(+) Actinomycetaceae*	*(−) Bilophila, Dysosmobacter*
Cerebellum functional network	*(+) Anaerotignaceae, (−) Eggerthellaceae*	*(+) Alloprevotella, Anaerotignum, CAG-177, Dysosmobacter Faecalicoccus, Intestinimonas, Limivicinus, Lactococcus, Lawsonibacter; (−) Faecalicoccus*
Visual functional network	*(+) Campylobacteraceae, Desulfovibrionaceae, Rikenellaceae Tannerellaceae,* UBA932	*(+) Alistipes, Angelakisella, Campylobacter, Cryptobacteroides, Eisenbergiella, Enterocloster, Hungatella, Parabacteroides, Phascolarctobacterium*
Sensorimotor functional network	*(−) Anaerotignaceae*	*(+) Collinsella; (−) Alloprevotella, Anaerotignum, CAG-177, CAG-485, Dysosmobacter, Eisenbergiella, Intestinimonas, Lactobacillus, Lawsonibacter, Limivicinus*
Auditory functional network	*–*	*(+) Blautia; (−) Peptostreptococcus, Ruthenibacterium*
Default mode functional network	*(+) Butyricicoccaceae, Lachnospiraceae*	*(−) Eubacterium*
Salience functional network	*(−), Akkermansiaceae, Pasteurellaceae, Veillonellaceae*	*(+) Limousia, (−) Akkermansia Alloprevotella, Anaerotruncus, Desulfovibrio, Lactobacillus, Lactococcus, Phascolarctobacterium, Veillonella*
Basal ganglia functional network	*–*	*(−) Ligilactobacillus, Ruthenibacterium*

Spearman’s rank correlation was performed between gut microbiota taxa and resting-state functional networks of piglets at weaning. Statistically significant correlations (*p* > 0.05) found between the relative abundance of individual taxa at the family and genus level and eight different functional networks are shown. (+) indicates a positive correlation and (−) indicates a negative correlation.

## 4 Discussion

In the current study, we demonstrated that the maternal supplementation of DHA during the perinatal stages led to the modulation of the gut microbiota of piglets at weaning. Maternal DHA supplementation significantly increased the alpha-diversity of gut microbiota in piglets, and some differences (Unweighted UniFrac) in the beta-diversity were present compared to the control piglets, as well as changes in the abundance of various taxa at the phylum, family, and genus level. Interestingly, such changes in microbial diversity and abundance were significantly correlated to the expression levels of tight junction proteins in the intestines, as well as the activation of different resting-state functional networks in piglet brains, suggesting that maternal DHA supplementation may influence offspring’s gut-brain axis via changes in epithelial tight junction status and functional connectivity at weaning.

Higher microbial richness and diversity, as well as significant alterations in specific microbial taxa were present in piglets born to DHA supplemented sows, indicating the role of maternal DHA intake during the perinatal period in shaping the microbiome of offspring. This is consistent with several human and animal studies reporting changes in offspring gut microbiota following maternal intake of DHA or fish oil (32, 33, 55, 56). For example, a recent study using a sow-piglet model reported that maternal fish oil supplementation (30 g/day) during late gestation and lactation increased Faith’s phylogenetic diversity, and the relative abundance of *Acidobacteria* in piglet fecal microbiome at 21 days of age (55). We also observed a trend of depletion of *Firmicutes* in piglets born to DHA-supplemented sows, which is in line with studies identifying decreased *Firmicutes* or *Firmicutes* to *Bacteroidetes* ratio following omega-3 PUFA or fish oil supplementation (57–59). Beneficial bacteria such as*, Bifidobacterium* was also enriched in DHA piglets compared to control piglets. Similarly, increased levels of *Bifidobacterium* is well documented in infants born to breastfeeding mothers that received fish oil supplementation (31), or adhered to dietary guidelines of 2–3 servings of fish per week during pregnancy (60).

The shaping of the infant gut microbiome relies heavily on maternal microbial transfer (61), suggesting that the changes observed in the piglet microbiome in our study may be partly attributable to DHA-induced alterations in the sow microbiome. This is further evidenced by previous findings showing that dietary supplementation of fish oil during late gestation and lactation leads to increases in the Simpson index of sow fecal microbiome (62). The vertical transfer of microbiota to the infant may occur through multiple pathways (61). Recent evidence on the presence of microorganisms in cord blood and amniotic fluid (63) of newborns suggests that the transmission of microbiota begins in the uterine environment (64), followed by other sources such as the birth canal during delivery (65) and exposure to maternal skin and oral sources (61). Breastmilk is one of the most important routes of maternal transfer to the infant (66, 67), constituting 27.7% of the infant gut microbiome within the first 30 days post birth (68). The existence of the entero-mammary pathway has been proposed in sows, by the shared bacterial genera across different body sites during farrowing, especially with a higher level of similarity between fecal and colostral samples compared to the blood (66). Alternatively, the changes found in piglet gut microbiome may have been driven by other factors influenced by DHA supplementation, such as inflammatory mediators (56) or milk components linked to metabolic pathways (62, 69). Since dietary supplementation of fish oil in sows can elevate the concentration of DHA in both the colostrum and piglet plasma (55), these changes could have resulted from maternal transfer, or occurred directly in the piglets due to increased DHA availability. It is important to note that this study did not include direct measurements of the sow’s microbial changes, and thus the suggested mechanisms of DHA-induced changes in the piglets remain speculative. Further studies including maternal microbial data from different sources such as colostrum, vaginal or feces will be necessary to confirm the specific pathways through which DHA induced microbial shaping in the offspring.

The gut microbiota interacts with the mucosal and the epithelial layer of the intestine to regulate gut permeability (52). Increased permeability induces leaky gut, allowing the translocation of pathogenic microorganisms such as endotoxins into the bloodstream, thereby promoting inflammatory responses (70). Thus, the regulation of the gut microbiome is integral to maintaining gut health. The beneficial effects of PUFA and fish oil in intestinal integrity are widely reported (55, 62, 67, 71). In humans, plasma DHA is inversely correlated with fecal zonulin levels (71), a marker for gut permeability. Sows receiving fish oil supplementation during late gestation and lactation has significantly lower concentrations of plasma zonulin (62) and another study showed that piglets born to fish oil supplemented sows have significantly lower levels of endotoxin and serum zonulin at 28 days of age (55). Also, supplementing piglets with fish oil increases the levels of tight junction proteins in the jejunum and ilium (72). Tight junction proteins, including claudins, occludins, and zonula occludens-1 (ZO-1), provide a physical barrier against pathogens by sealing the paracellular space between epithelial cells (52, 70). Our results showed that maternal DHA supplementation significantly increased the expression level of claudin-1 in piglets, indicating its potential for improving structural barrier integrity. Furthermore, the levels of tight junction proteins in piglet jejunum were significantly correlated with specific microbial taxa, supporting the idea that certain gut microbes are closely linked to modulations in the epithelial tight junction. Overall, various taxa including *Acidobacteriota, Chloroflexota, Kineothrix, Akkermansiaceae, Akkermansia, Veillonella,* and *Veillonellaceae* were significantly correlated to both claudin-1 and occludin. Notably, *Akkermansia is* a genus well-recognized for its role in improving gut barrier function. A recent study identified that *Akkermansia muciniphila* facilitates the expression of a transcription factor cAMP-responsive element-binding protein H (CREBH), which attenuates ER stress and upregulates tight junction protein expression in the gut (73). The link may also be explained by *Akkermansia*-derived EVs that signal the AMPK pathway to facilitate the expression of tight junction proteins (74). In a mice model with intestinal damage induced with bisphenol A, DHA-enriched phosphatidylserine treatment restored the levels of tight junction proteins in the jejunum, accompanied by an increased abundance of *Akkermansia muciniphila* (75). *Veillonella* has also been reported to be positively correlated to claudin levels in rheumatoid arthritis patients (76). Moreover, claudin was correlated to *Bifidobacteriaceae* and *Bifidobacterium,* which have shown protective effects on tight junction integrity and reduce permeability and inflammation (77–79). We believe that the microbial changes in the piglets following maternal DHA supplementation may have interacted closely with tight junction proteins in their guts. Additional studies including functional barrier assays will be necessary to better understand the relationship between DHA-induced gut microbiota and tight junction proteins in offspring.

During the perinatal stages, DHA is widely used as a supplement due to its known benefits on cognition and neurodevelopment of infants. Since the offspring’s endogenous synthesis does not meet the demand for large amounts of DHA during the period of rapid brain development (80), maternal intake of DHA appears to be of particular importance. In our previous study, DHA supplementation of sows increased exploratory behaviors, memory function and affected brain functional organization of their piglets at weaning (7). Additional neurochemical evaluations of these piglets identified changes in serotonin levels in specific brain regions that may lead to improved brain function and development (81). In recent years, an increasing number of studies have shown the significance of the gut-brain axis in different inflammatory, endocrine, and neuroanatomic pathways. It is believed that the gut microbiota acts as a mediator, playing a key role in cognitive function and behavior. This is pronounced by the shared critical period of development between the gut and the brain, offering an important opportunity for intervention to improve cognitive development. Indeed, evidence demonstrates a link between microbial diversity and cognition across the lifespan, ranging from infancy (82), midlife (14, 83), and in older adults with (84) or without cognitive impairment (85, 86).

In the current study, we found significant associations between the gut microbiota and resting-state functional networks in piglets. While the links between specific functional connectivity and gut microbiota remain to be investigated, our results are consistent with the idea that gut microbiota shaping has a significant effect on functional connectivity (87), including that of healthy infants (88). One of the most frequently identified networks in association with the gut microbiota is the salience network (89). The regulation of the salience network is of great importance, as an overactive or underperforming salience network has been implicated in various cognitive deficits (90). In this study, the activation of the salience network showed negative correlations with the number of observed features. Similar findings have been reported in a previous study with healthy infants that identified a negative association between microbiome diversity and the functional connectivity between the anterior cingulate cortex and the right anterior insula, which plays a role in the salience control center (88). Germ-free mice displayed hyperconnectivity with poor organization compared to mice with normal gut microbiome (87), further supporting the regulatory role of the gut microbiome in brain functional connectivity. In addition, Shannon’s diversity was positively correlated to the activation of cerebellum network and visual network. In males, increased Shannon’s index has been linked to higher GMV in the cerebellum (91), and the visual network was identified as a mediator between the gut microbiota alpha diversity and working memory and attention (92). In infants, positive associations between alpha diversity of microbiome and functional connectivity of brain areas linking the auditory, visual and somatosensory cortices were present (88). We also observed significant correlations between the different functional networks and the relative abundance of microbiota at different taxonomic levels, including those reported in previous studies, such as *Bacteroidota, Ruminococcus, Blautia, Collinsella, and Enterococcus* (89). Together this represents a strong link between gut microbiota and brain functional connectivity of developing piglet brains.

Nevertheless, it is important to note that correlational findings need to be approached with caution. Our current correlation data does not provide information on the directionality of the relationship between gut microbiome and brain functional outcomes and additional experiments will be needed to understand the mediation of gut microbiome on the gut-brain axis. Also, heterogeneity exists in current research exploring the links between the gut microbiota and brain connectivity, driven by factors such as methodological differences (89) and selection biases (92) that may limit the reproducibility of the results. Moreover, while many bacterial populations have been identified to be correlated with general behavior traits (93), the correlations are less frequently identified when examining distinct cognitive traits, such as learning and memory (94). This strongly evidences the high complexity and multi-layered characteristic of the communication between the gut and the brain that influences specific cognitive function. Therefore, these correlations require further interpretation through rigorous, standardized methods and in-depth mechanistic studies. Furthermore, the microbiome data in the current study were analyzed by 16S rRNA gene sequencing. While 16 s rRNA sequencing is a widely used and cost-effective method for microbiome analysis, it has important limitations such as inability to directly assess functional profiles (95, 96). Thus, future studies will benefit from including metagenomic shotgun sequencing data for a more robust functional interpretation of the microbiome changes.

In conclusion, maternal DHA supplementation has profound effects on the gut microbiome of offspring, leading to improved gut integrity at weaning. We observed links between changes in gut microbiota with specific resting-state functional network activation in the piglet brains, suggesting the role of microbiota in regulating the gut-brain axis during the perinatal period. While existing literature links gut microbiota to cognitive impairment or neurodegenerative diseases, the role of gut microbiota in cognitively healthy subjects remains to be elucidated. Therefore, the findings of this study using a highly translatable, healthy pig model help better understand the role of gut microbiota in early-life brain development, underscoring the potential of maternal nutritional intervention to program the gut-brain axis.

## Data Availability

The original contributions presented in the study are publicly available. This data can be found here: https://www.ncbi.nlm.nih.gov/bioproject/PRJNA1405157.
