# Supplementary material for: Maternal docosahexaenoic acid supplementation shapes offspring gut microbiota to modulate the gut-brain axis in a sow-piglet model
Source: Front Nutr. 2026 Apr 13;13:1776896. doi: 10.3389/fnut.2026.1776896 (PMC13110974; doi:10.3389/fnut.2026.1776896)
Supplement: Supplementary file 1 [file Table_1.DOCX]

Supplementary Material

**Supplementary Figure 1. ADONIS Test Results of Unweighted UniFrac of Piglet Gut Microbiota at Weaning.**

|  | **Df** | **SumsOfSqs** | **MeanSqs** | **F.Model** | **R2** | **Pr(>F)** |
| --- | --- | --- | --- | --- | --- | --- |
| **Treatment** | 1 | 0.388746 | 0.388746 | 3.906861 | 0.152361 | 0.001 |
| **SowID** | 1 | 0.088773 | 0.088773 | 0.892161 | 0.034793 | 0.572 |
| **Treatment:SowID** | 1 | 0.083884 | 0.083884 | 0.843030 | 0.032877 | 0.675 |
| **Residuals** | 20 | 1.990070 | 0.099503 | NaN | 0.779969 | NaN |
| **Total** | 23 | 2.551474 | NaN | NaN | 1.000000 | NaN |

**Supplementary Figure 2. ADONIS Test Results of Weighted UniFrac of Piglet Gut Microbiota at Weaning.**

|  | **Df** | **SumsOfSqs** | **MeanSqs** | **F.Model** | **R2** | **Pr(>F)** |
| --- | --- | --- | --- | --- | --- | --- |
| **Treatment** | 1 | 0.086422 | 0.086422 | 1.200285 | 0.053784 | 0.263 |
| **SowID** | 1 | 0.037664 | 0.037664 | 0.523102 | 0.023440 | 0.848 |
| **Treatment:SowID** | 1 | 0.042720 | 0.042720 | 0.593317 | 0.026586 | 0.765 |
| **Residuals** | 20 | 1.440023 | 0.072001 | NaN | 0.896190 | NaN |
| **Total** | 23 | 1.606828 | NaN | NaN | 1.000000 | NaN |
